# TRP Channels: Recent Development in Translational Research and Potential Therapeutic Targets in Migraine

**DOI:** 10.3390/ijms24010700

**Published:** 2022-12-31

**Authors:** Eleonóra Spekker, Tamás Körtési, László Vécsei

**Affiliations:** 1 ELKH-SZTE Neuroscience Research Group, University of Szeged, Semmelweis u. 6, H-6725 Szeged, Hungary; 2 Faculty of Health Sciences and Social Studies, University of Szeged, Temesvári krt. 31, H-6726 Szeged, Hungary; 3 Department of Neurology, Faculty of Medicine, Albert Szent-Györgyi Clinical Center, University of Szeged, Semmelweis u. 6, H-6725 Szeged, Hungary

**Keywords:** migraine, neurogenic inflammation, pain, TRP channel, TRPV1, TRPV4, TRPM8, TRPA1, migraine therapy

## Abstract

Migraine is a chronic neurological disorder that affects approximately 12% of the population. The cause of migraine headaches is not yet known, however, when the trigeminal system is activated, neuropeptides such as calcitonin gene-related peptide (CGRP) and substance P (SP) are released, which cause neurogenic inflammation and sensitization. Advances in the understanding of migraine pathophysiology have identified new potential pharmacological targets. In recent years, transient receptor potential (TRP) channels have been the focus of attention in the pathophysiology of various pain disorders, including primary headaches. Genetic and pharmacological data suggest the role of TRP channels in pain sensation and the activation and sensitization of dural afferents. In addition, TRP channels are widely expressed in the trigeminal system and brain regions which are associated with the pathophysiology of migraine and furthermore, co-localize several neuropeptides that are implicated in the development of migraine attacks. Moreover, there are several migraine trigger agents known to activate TRP channels. Based on these, TRP channels have an essential role in migraine pain and associated symptoms, such as hyperalgesia and allodynia. In this review, we discuss the role of the certain TRP channels in migraine pathophysiology and their therapeutic applicability.

## 1. Migraine and Other Headache Disorders

### 1.1. Headache Disorders

The term headache disorder covers a wide range of neurological conditions that cause painful symptoms in the head and can vary in frequency and intensity. Adequate treatment is possible only on the basis of the correct diagnosis. Different types of headaches can be divided into two main groups, primary and secondary headaches.

Primary headaches are one of the most common neurological disorders, with a wide range of life-long manifestations. It is caused by the over-functioning of the pain-sensitive structures of the head or related problems. In this case, the headache is not a symptom of an underlying disease or condition. The most common types of primary headaches include tension-type headache, cluster, migraine, or other trigeminal autonomic cephalalgias [1,2].

According to the International Classification of Headache Disorders, a secondary headache is defined as a headache that occurs in close temporal relation to another disorder known to cause a headache or meets other criteria for a cause-and-effect relationship with the disorder. Possible causes of secondary headaches include brain aneurysm or tumor, infections of the central nervous system (encephalitis, meningitis), neck or brain injury, and idiopathic intracranial hypertension, etc. [3].

Tension-type headache is a common headache disorder that affects around 42% of adults worldwide. These headaches often cause mild-to-moderate pain around the head, face, or neck. The pain is commonly bilateral, described as aching or pressure, as “a band around the head”, or vice-like. It usually is not associated with visual disturbances, nausea, or vomiting, and physical activity does not make the headache pain worse. Triggers of tension headache attacks include stress, alcohol, dental problems, smoking, caffeine, and fatigue. For the treatment of tension-type headaches, over-the-counter analgesics such as paracetamol, ibuprofen, nonsteroidal anti-inflammatory drugs (NSAIDs), or aspirin are effective treatment strategies. If tension-type headaches are frequent and long-lasting and over-the-counter pain relievers are ineffective, tricyclic antidepressants (amitriptyline) and beta-blockers may be a good choice for headache treatment [4].

Cluster headache is an extremely grievous trigeminal autonomic cephalalgia. The pain is unilateral and localized around the orbital, supraorbital, or temporal regions. The headache attack lasts for 15 to 180 min and is associated with lacrimation, eyelid edema, nasal congestion, or rhinorrhea. Cluster headaches usually happen every day, in bouts lasting several weeks or months at a time (typically 4 to 12 weeks) before they subside. People often feel restless and agitated during an attack because the pain is so intense. The goal of therapy is to decrease the severity of pain, shorten the attack period, and prevent headaches. Acute treatments are oxygen, triptans, and local anesthetics, while preventive therapies are calcium channel blockers, corticosteroids, lithium carbonate, etc. [5,6].

Simultaneous activation of the trigeminal and autonomic nervous systems is a common feature of all trigeminal autonomic cephalalgias. It produces the clinical picture of short-lasting, strictly unilateral headache attacks with ipsilateral autonomic symptoms such as lacrimation, ptosis, nasal congestion or rhinorrhea, and conjunctival injection [5].

This review focuses on migraine.

### 1.2. Migraine

A migraine is a primary headache characterized by attacks of a moderate to severe headache that last for 4–72 h, a unilateral, throbbing headache accompanied by a variety of other symptoms, such as phono- and photophobia, nausea, and vomiting [7]. These associated symptoms notably affect the quality of life and entail economic costs [8]. Recently, migraine was identified by the Global Burden of Disease study as the most disabling neurological disorder [9]. Migraine attacks can also be accompanied by an aura phenomenon, which consists of visual, sensory, or speech symptoms. These appear gradually, last up to 60 min, and are completely reversible [10].

Many internal and external stimuli, such as stress, weather, and consumption of certain foods and alcohol, can induce migraine attacks [11,12]. Marmore de Lima et al. found that odors, especially perfume and cigarette smoke, were the second most common triggers for migraine attacks [13]. Moreover, Nicoletti et al. reported that high-dose ethanol causes neurogenic inflammation, which can be induced by the activation of transient receptor potential vanilloid 1 (TRPV1) and the release of neuropeptides in trigeminal nociceptors [14].

The generation of migraine headaches remains elusive, but the activation and sensitization of the trigeminal system are important during migraine attacks [15]. As a result, a number of proinflammatory neuropeptides and neurotransmitters, including calcitonin gene-related peptide (CGRP), substance P (SP), neurotensin, and hemokinin A are released from the sensory fibers and cause a series of inflammatory tissue responses [16,17,18,19].

Glutamate is an essential neurotransmitter in the central nervous system and plays a role in nociceptive processing in both physiologic and pathologic conditions. Activation of primary nociceptive afferents leads to glutamate release. Increased levels of glutamate have been observed in blood and cerebrospinal fluid in migraine patients both interictally and ictally, which supports its role in migraine [20]. Important endogenous regulators of glutamatergic neurotransmission include certain metabolites of the kynurenine pathway formed during the catabolism of tryptophan. The N-methyl-D-aspartate receptor (NMDA) antagonist kynurenic acid (KYNA) is one of the end products of this path, which can inhibit the activation of the trigeminal system [21,22]. KYNA and its derivative can block the nitroglycerin (NTG)-induced release of c-fos (an activation marker) [23] and CGRP [24], as well as the electrical stimulation of trigeminal ganglion-induced PACAP overexpression [22]. Aside from the translational investigations, clinical studies prove the role of the kynurenine pathway in the pathophysiology of primary headaches. Alterations of kynurenine metabolism were shown in the plasma of migraineurs [25] and patients suffering from cluster headache [26].

The activation of nociceptors innervating the vascular system of the brain and meninges may also be responsible for the development of headache [18,27,28]. Inflammatory mediators can enhance transient receptor potential (TRP) channels through different signaling pathways, which contributes to the maintenance of inflammatory hyperalgesia [29,30,31]. These changes may be behind the long and severe headache that develops during a migraine. In addition to headaches, 25–30% of patients also experience an aura phenomenon during some attacks, which is manifested by temporary visual, sensory, language, or brainstem disturbances [32]. According to our current knowledge, cortical spreading depression (CSD) is responsible for the aura [33]. CSD is a slowly propagating wave of transient neuronal and glial depolarization. Associated with this wave of depolarization characteristic of CSD are local ionic shifts and the release of neurotransmitters. This includes an increase in extracellular potassium and a decrease in extracellular sodium, chloride, and calcium [34]. Studies have reported that CSD induces macrophage activation, mast cell degranulation, and dural vessel dilation, and all of these changes can induce a headache [35,36].

Thanks to the advances in neuroimaging, the view that the hypothalamus plays an important role in the development of the most common symptoms after the onset of the headache phase of a migraine attack is increasingly accepted. It is possible that the hypothalamus can regulate descending modulation of trigeminovascular processing in a state-dependent manner. It is supported by the activation of several hypothalamic nuclei in response to dural nociceptive stimulation. Moreover, the frequent occurrence of hypothalamic-related disturbances, such as altered appetite regulation, sleep–wake states, and nociceptive processing further supports the role of the hypothalamus in migraine [32]. Recent findings suggest that spontaneous oscillations of the complex brain networks, including the hypothalamus, brainstem, and dopaminergic networks, cause changes in the activity of subcortical and brainstem areas, thereby changing sensitivity thresholds and triggering headache attacks [37].

Despite the significant socio-economic burden of migraine, the effective treatment of migraine remains unresolved. Differentiating migraine from other headache disorders is important. It can mean faster relief through more targeted treatments based on the type of headache. The ideal migraine preventive treatment should be effective and well tolerated, with only a few mild side effects and without contraindications. Current drugs have not been able to achieve these goals. The most frequently used treatments are triptans and NSAIDs [38,39], but they are not always effective. Moreover, triptans are contraindicated in people with cardiovascular disease, and their excessive use can lead to medication overuse headache (MOH) [7,40,41]. To eliminate these negative properties, ditans, 5-HT_1F_ receptor agonists, were developed, but currently, only one ditan can be used in the clinic as an antimigraine agent and does not exceed the effectiveness of triptans.

The latest antimigraine drugs include monoclonal antibodies targeting CGRP or its receptor. According to the available data, monoclonal antibodies targeting the CGRP pathway appear to be effective and well tolerated and showed fewer side effects in clinical trials when compared to existing treatments. They also offer a lower risk of drug interactions [42]. However, objective biomarkers of treatment response are still lacking, and the long-term safety risks of these medicines are still unknown.

Therefore, further investigations are needed to understand the underlying mechanisms of migraine and discover new therapeutic options, which may improve diagnosis and provide more personalized treatment for this condition.

## 2. Transient Receptor Potential Channels

**Figure 1:**
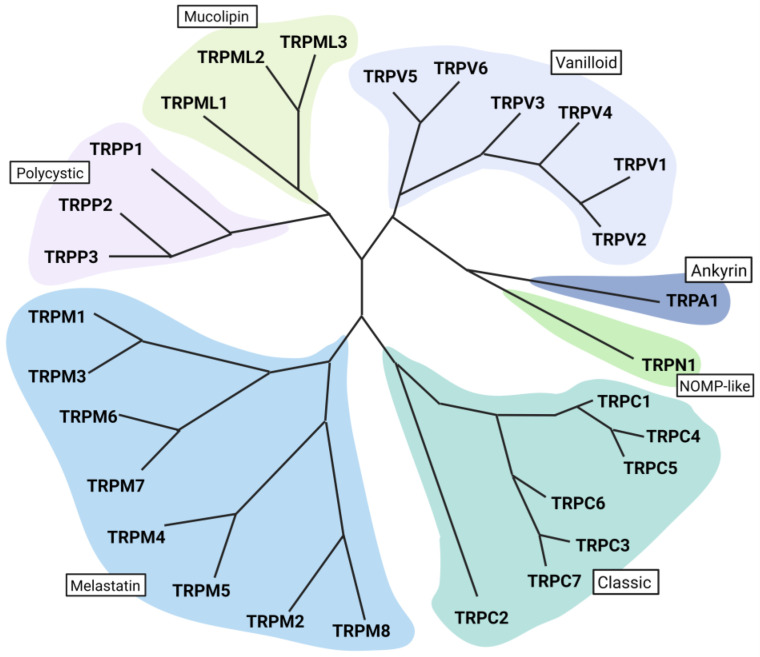
The transient receptor potential family encodes integral membrane proteins that function as ion channels. TRP channels are divided into seven subfamilies based on their homology of amino acid sequences. Most TRPs are nonselective cation channels and can be activated in a variety of ways, ranging from ligand binding, voltage, and temperature changes to covalent modification of nucleophilic amino acids. Activated TRP channels cause the depolarization of the cell membrane and are involved in the transcellular transport of many cations (Ca^2+^, Mg^2+^), and they also contribute to the functioning of endosomes and lysosomes.

Mammalian TRP channels are divided into seven subfamilies based on their homology of amino acid sequences: canonical or classic (TRPC), vanilloid (TRPV), melastatin (TRPM), nonmechanoreceptor potential C (NOMP-like, TRPN1) polycystin (TRPP), mucolipin (TRPML), and ankyrin (TRPA) [43,44] (Figure 1).

TRP channels respond to a wide spectrum of physical and chemical stimuli, including temperature, stretch/pressure, chemicals, oxidation/reduction, osmolarity, and pH [45,46]. Activation of TRP channels causes cations to cross the membrane and depolarize cells, leading to a wide range of cellular responses [47,48]. TRP channels play a variety of physiological roles, and they are involved in several diseases affecting the peripheral (PNS) and central nervous system (CNS) [49,50]. The assumption that they play a role in migraines is based on their expression on meningeal nociceptors and their response to several endogenous and exogenous stimuli [51,52].

Many TRP channels are expressed in nociceptive sensory neurons, and these TRP channels are involved in the generation and transmission of pain, so they may represent a new therapeutic option for pain relief [53,54]. The use of TRP channels in migraine therapy is supported by the clinical success of CGRP receptor antagonism, as activation of TRP channels can induce CGRP release [55,56].

Evidence suggests that sex hormones directly regulate nociceptor activity at the transcriptional, translational, and functional levels. Sex hormones can modulate the expression of TRP channels via their channel activity or by activating intracellular signaling pathways, which contribute to the sex dimorphism that occurs in migraine [57,58].

To date, several studies have been published on TRP channels and migraine, supporting the role of these channels in the activation of meningeal nociceptors [51,55,59,60,61,62]. In this review, we summarized the TRP channels that—according to our current knowledge—are involved in migraine pathophysiology and the therapeutic potential of these channels in the treatment of primary headaches.

### 2.1. Transient Receptor Potential Vanilloid 1 (TRPV1)

#### 2.1.1. Characterization of TRPV1 and Role in Pain and Headaches

**Figure 2:**
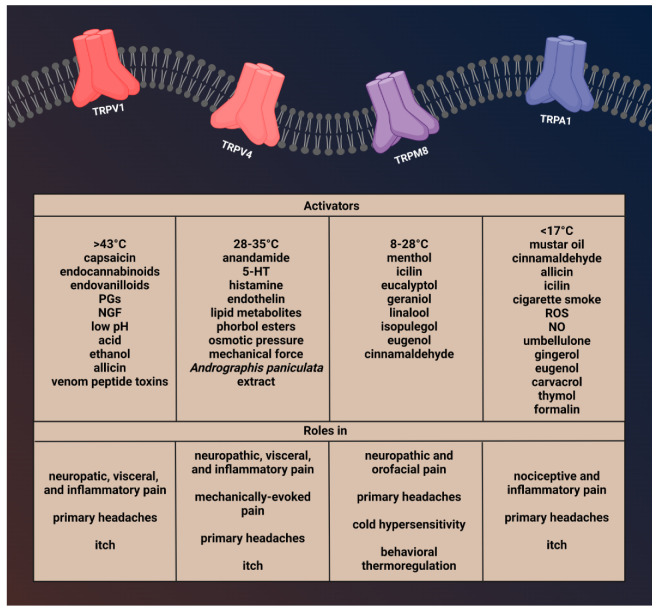
Activators and function of transient receptor potential channels involved in migraine.

**Figure 3:**
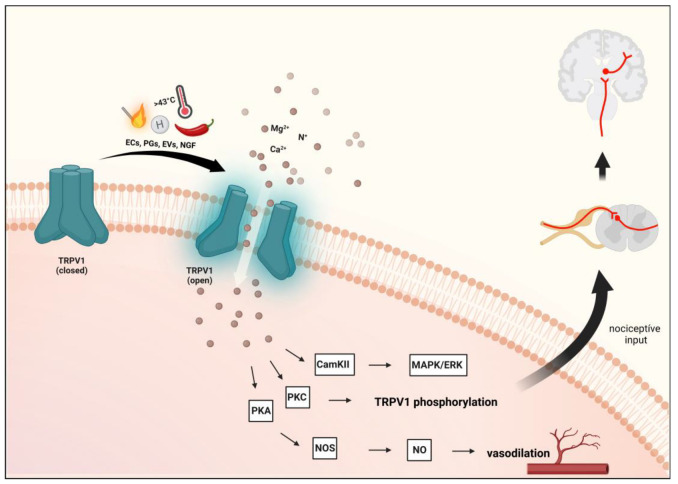
Transient receptor potential vanilloid 1 receptor activation. Activation of TRPV1 and the resulting influx of cations can further activate voltage-gated ion channels to generate action potentials to be required for pain or itch signaling. Several inflammatory mediators lower the activation threshold of TRPV1 via phosphorylation mainly through the activation of the cAMP-dependent protein kinase A (PKA) pathway. Furthermore, protein kinase C (PKC)-dependent cascade is also involved. TRPV1: transient receptor potential vanilloid 1 receptor, ECs: endocannabinoids, EVs: endovanilloids, PGs: prostaglandins, NGF: nerve growth factor.

One of the first TRP channels to be investigated was TRPV1, which is a nonselective cation channel responsive to high temperature (>43 °C) and capsaicin (the main pungent ingredient in “hot” chili peppers) [63], which have been shown to activate sensory nerves and induce neurogenic inflammation (NI) [64]. TRPV1 is also sensitive to endocannabinoids, endovanilloids, nerve-growth factor (NGF), and prostaglandins (PGs), which may be relevant for migraine [65]. The hydrogen ion, acid, or low pH also can activate the TRPV1 channel [66] (Figure 2 and Figure 3).

Several studies have shown that approximately 40–50% of trigeminal sensory neurons express TRPV1 [67]. Furthermore, it is expressed in small amounts in the hypothalamus, hippocampus, entorhinal cortex, raphe nucleus, and the periaqueductal gray matter (PAG) [68,69,70,71]. TRPV1 receptor is present in small and medium-diameter neurons of the dorsal ganglion (DRG) and trigeminal ganglia (TG), colocalized with CGRP and SP in the latter [63]. In addition, TRPV1 and NMDAR are coexpressed in the TG [72]. However, TRPV1 has also been described in brain areas that are not associated with pain or heat sensations, such as the ventral tegmental area or the striatum [73,74].

Upon activation of TRPV1, CGRP and SP are released, causing vasodilation and triggering NI in the meninges [75,76]. Furthermore, in sensory neurons, activation of TRPV1 by NO leads to peripheral sensitization and nociception [77].

After tissue damage, endogenously released inflammatory mediators such as bradykinin, serotonin (5-HT), PGs, or histamine can influence TRPV1 activity, mainly indirectly through the stimulation of their receptors and the generation of second messengers [78,79]. TRPV1 is a molecular component of pain sensation and modulation [80]. Activation of TRPV1 and the resulting influx of cations can further activate voltage-gated ion channels to generate action potentials required for pain or itch signaling [67]. The sensitization and endogenous regulatory pathways of TRPV1 can exert their effects through the phosphorylation sites of protein kinases C (PKC) and A (PKA) and Ca^2+^/calmodulin-dependent kinase II (CAMKII) [30,81]. Prolonged or repeated activation of TRPV1 prompts a desensitization or inhibition process [79], thereby losing the sensitivity to capsaicin and other chemical agonists, further reducing the sensitivity to heat [82].

#### 2.1.2. Preclinical Data

Caterina et al. reported that TRPV1−/− mice responded normally to noxious mechanical stimuli. At the same time, pain behavior induced by vanilloids was not observed. In these knock-out mice, painful heat perception was reduced, and little thermal hypersensitivity was detected [80]. Based on these, it can be said that TRPV1 is essential for the sensation of pain and thermal hyperalgesia. Moreover, Davis and colleagues have found that TRPV1 has a major role in the sensitization to thermal stimuli during inflammation but not in the normal sensation of noxious heat [83]. With repeated administration of capsaicin, pain receptors can become more sensitive to chemical stimuli, so that in desensitized animals, mustard oil or xylene does not cause pain or inflammatory response [63,84]. Intraplantar injection of Complete Freund’s adjuvant caused local inflammation and increased the number of TRPV1-positive cells in the DRG [85]. In guinea pig, ethanol—a trigger of headache—can activate TRPV1 on primary afferent neurons, thereby leading to neurogenic inflammation and CGRP-mediated vasodilation [14]. In the nitroglycerin model of migraine, chronic intraperitoneal administration of ghrelin can reduce mechanical and thermal hypersensitivity and the associated increased CGRP and TRPV1 mRNA expression in the TG [86]. In another animal model of migraine, after application of inflammatory soup on the dura mater, the TRPV1 immunoreactivity is increased in the dorsal horn of the spinal cord, which was modulated by sumatriptan [87].

#### 2.1.3. Clinical Data

In headache and pain research, TRPV1 is a very promising target for the development of new analgesics for the treatment of inflammatory and neuropathic pain.

Capsaicin is a TRPV1 agonist that helps relieve pain when used in the right amount and frequency [88]. Creams containing capsaicin have long been used to treat postherpetic neuralgia, diabetic neuropathy, and rheumatoid arthritis [89]. The results of a single-blinded placebo-controlled study showed the topical administration of capsaicin on painful scalp arteries can reduce or eliminate pain in migraine patients. It can also prevent mild migraine attacks [90]. Another study investigated the effect of capsaicin on chronic migraineurs. It was found that repeated intranasal capsaicin treatment reduced migraine attacks by 50–80% in these patients [16]. This is probably due to the fact that intranasal administration of capsaicin locally desensitizes the trigeminal nerve, resulting in a decrease in CGRP. Other studies observed a significant reduction in cluster seizures in patients who received capsaicin rather than placebo treatment [91,92]. However, long-term use of capsaicin may increase the risk of skin carcinogenesis, especially when used in combination with tumor promoters [93].

Resiniferatoxin (RTX), a chemical irritant, was originally isolated from *Euphorbia resinifera Berg* [94]. RTX is an ultrapotent capsaicin analog, more potent than capsaicin, and is currently being developed as a sensory neuron desensitizer. RTX causes a prolonged calcium influx that causes cytotoxicity and death only in sensory neuronal cell bodies that express the TRPV1 in the DRG [63,95]. A large clinical trial investigated the effect of intravesical RTX in patients with interstitial cystitis, but desensitization of TRPV1-expressing afferents did not provide a clear improvement compared to placebo [96]. RTX is being studied in clinical trials for pain relief in advanced cancer patients and for the treatment of moderate to severe knee pain caused by osteoarthritis. Preliminary results suggest that RTX exerts long-lasting analgesic effects by causing cell death of TRPV1-positive nociceptor neurons [97]. The intra-articular injection of RTX is also under development, and its effectiveness and reliability are being tested in a Phase III study.

Civamide (Zucapsaicin) is a cis-isomer of capsaicin and excites and desensitizes C-fibers via TRPV1 on nociceptive neurons. Intranasal administration of civamide inhibits the neuronal release of excitatory neurotransmitters such as CGRP and SP from the trigeminal plexus centrally into meningeal and dural blood vessels [98], thereby reducing vasodilation, plasma extravasation, and histamine and 5-HT release. As a result, NI does not develop and can alleviate cluster headaches [99]. Diamond et al. investigated the effect of civamide for the treatment of a single migraine headache, with or without aura, of moderate to severe pain. After 2 h from treatment, 55.6% of patients had a decrease in pain severity, with 22.2% of patients being pain-free, while after 4 h, 72.7% of patients reported reduced pain intensity, and 33.0% of patients felt no pain [100]. However, initial peripheral neuropeptide release causes unpleasant side effects, such as nasal burning, lacrimation, and rhinorrhea in most patients [100].

A TRPV1 antagonist, AMG-8562, effectively blocks capsaicin- and anandamide-induced receptor activation but not heat-induced TRPV1 activation [101]. However, an unexpected effect of AMG-8562 is to enhance proton-induced Ca^2+^ influx in TRPV1-expressing cells [101].

Another competitive TRPV1 receptor antagonist is SB-705498, which results in rapid and reversible inhibition of capsaicin-, acid (pH 5.3)-, or heat (50 °C)-mediated activation of human TRPV1 [102]. In a study by Chizh et al., a single oral dose of 400 mg SB-705498 significantly reduced capsaicin-induced cutaneous pain and flare compared with the placebo group [103]. However, SB-705498 was inferior to placebo against migraine headache, photophobia, and phonophobia in a Phase II clinical trial [52]. While several TRPV1 antagonists have progressed to clinical trials, they have failed to advance to Phase II trials due to adverse side effects such as hyperthermia (AMG-517, AZD-1386, ABT-102, MK-2295) or loss of thermal pain sensitivity (ABT-102, MK-2295) [104].

Currently, only two TRPV1 antagonists have been reported to enter Phase III trials for atopic dermatitis (PAC-14,028) and dry eye syndrome (SYL-1001) [105] Hopefully, future clinical studies with TRPV1 receptor antagonists provide an answer as to the role of TRPV1 in inflammatory and neuropathic pain syndromes.

Genetic studies suggest that the TRPV1 gene is linked to migraines [106,107]. Yakubova et al. found that the frequency distribution of SNP 1911A > G AA, AG, and GG variants in the TRPV1 gene is differently associated in episodic and chronic migraine patients than in healthy individuals [107]. Thus, the detection of these gene variants may serve as markers of protection against migraine chronicity and may offer an opportunity for personalized treatment of migraine patients.

### 2.2. Transient Receptor Potential Vanilloid 4 (TRPV4)

#### 2.2.1. Brief Description of TRPV4 and Its Role in Pain and Headache

**Figure 4:**
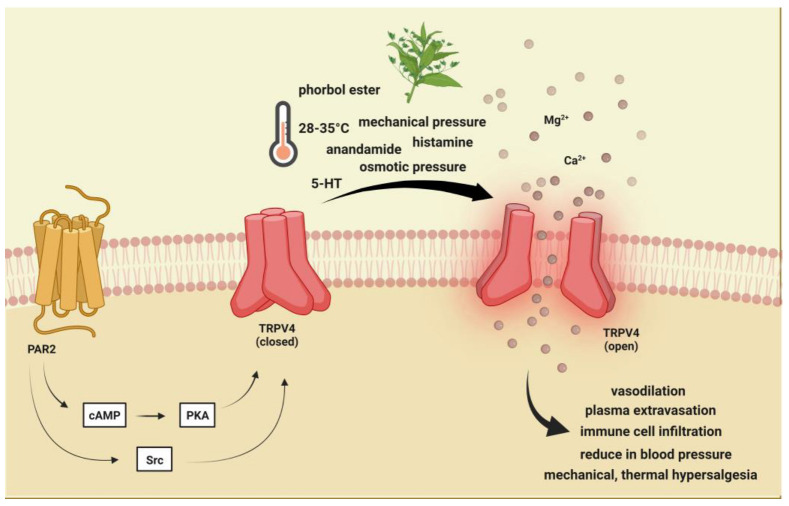
Transient receptor potential vanilloid 4 receptor activation. TRPV4 is activated by moderate heat (>24 °C to 27−35 °C), low pH, endocannabinoids, lipid metabolites, osmotic pressure, and phorbol ester and plant-derived compounds. PAR-2 activation may indirectly sensitize (via PKA, PKC, and PLC) TRPV4, thereby contributing to mechanical allodynia and thermal hyperalgesia. TRPV4: Transient receptor potential vanilloid 4, 5-HT: serotonin.

TRPV4 is a polymodal cation channel activated by moderate heat (>24 °C to 27−35 °C), low pH, endocannabinoids, lipid metabolites, osmotic pressure, and phorbol ester and plant-derived compounds [108,109,110]. It plays a crucial role in mechanical-, thermal-, and chemical-induced pain sensitivity [111]. TRPV4 is also involved in the regulation of vascular tone and acute inflammatory signaling [112,113] and functions as part of the mechanosensory complex. Based on these, the functions and trigeminal localization of TRPV4 may fit some aspects of migraine, such as the characteristic throbbing pain that is aggravated by routine movements, coughing, or sneezing [52]. Another finding supporting the role of TRPV4 in migraine is that solutions applied to the surface of the dura mater that increase or decrease osmolarity can sensitize trigeminal afferents [114,115,116]. TRPV4-dependent pathways promote plasma extravasation and immune cell infiltration by increasing the release of some neuropeptides, including CGRP and SP, and thus are considered to be potentiators of neurogenic inflammation [117] (Figure 2 and Figure 4).

TRPV4 is widely expressed in various regions in the PNS and CNS, including immune cells, hippocampal neurons, nonpeptidergic, Aβ and Aδ fibers neurons of DRG, and peptidergic C fibers, where it coexpresses with TRPV1 [118]. Aside from the neurons TRPV4, is also present in nonmyelinating Schwann cells and satellita glial cells [119]. TRPV4 mRNA is expressed in TG, and in vitro investigations prove functional effects of receptor on trigeminal neurons [120]. Furthermore, TRPV4 shows colocalization with CGRP, SP, and protease-activated receptor 2 (PAR2) in rat sensory neurons [121]. In addition to PAR2, the role of TRPV4 in inflammation has also been associated with histamine and serotonin. Histamine- or serotonin-induced visceral hypersensitivity is significantly reduced when TRPV4 is blocked with siRNA, indicating TRPV4-dependent histamine- or serotonin-mediated response in sensory neurons [122].

#### 2.2.2. Preclinical Data

Experimental data proved that TRPV4 knock-out mice exhibit abnormal osmotic regulation and reduced nociceptive responses to pressure and hypertonic stimuli [118,123,124].

In an animal model, TRPV4 contributed to formalin-induced trigeminal pain response. TRPV4 is found in sensory neurons of the TG, which respond to formalin treatment with Ca^2+^ influx in a TRPV4-dependent manner. Thus, TRPV4 may play a key role in the prolonged, neuron-mediated tonic phase of pain behavior [125]. In a rat model of neuropathic pain, a taxol-induced peripheral neuropathy and mechanical hyperalgesia was reduced by inhibition of TRPV4 activation [126]. In a rodent model of neurogenic inflammation, inhibition of TRPV4 activation decreased the PAR2-mediated inflammatory processes in rat primary nociceptive neurons [127,128].

4α-phorbol-12,13-didecanoate (4α-PDD), a selective TRPV4 agonist, can interact directly with the TRPV4 channel at low concentrations [129,130,131]. In an in vivo model, the application of a hypotonic solution or 4α-PDD caused both facial and hind paw allodynia, possibly due to activation of TRPV4 in dural afferents. This assumption is supported by the fact that the administration of a TRPV4 antagonist was able to inhibit this headache-related behavior [132].

Another TRPV4 agonist is 5′,6′-epoxyeicosatrienoic acid, which mediates TRPV4 activation in response to hypoosmotic shock or mechanical stimulation [133,134,135].

It has been reported that bisandrographolide A (BBA), an extract from a Chinese herb (Andrographis paniculata), can selectively activate TRPV4. It is possible that BAA can affect the vascular smooth muscle due to the activation of TRPV4 and cause arterial dilation and reduce blood pressure [136].

GSK1016790A, a selective agonist of TRPV4, can activate TRPV4 in different cell types and elicit Ca^2+^-dependent increases in extracellular ATP levels. However, the mechanism of TRPV4 response to GSK1016790A is not very well known [137].

TRPV4 antagonist RN1734 was able to inhibit the TRPV4-induced cephalic and extracephalic allodynia in freely moving rats [132]. Although TRPV4 inhibition would be an interesting target for pain, deafness, incontinence, and osmoregulation, deficits were observed in TRPV4-knockout mice, which questions the clinical utility of TRPV4 antagonists [138,139,140]. In addition, the essential role of TRPV4 in central osmoregulation may also limit its therapeutic options.

Unfortunately, most of the results obtained from preclinical studies provide only indirect evidence for a link between TRPV4 and migraine. Further studies are needed to explore the potential role of TRPV4 in headache disorders.

#### 2.2.3. Clinical Data

Over the past decade, drug research has focused on the development of TRPV4 antagonists for therapeutic use. GlaxoSmithKline reported a series of TRPV4 antagonists, quinolines, and benzimidazoles, which failed to enter clinical trials.

To date, only one TRPV4 antagonist, GSK2798745, has entered clinical trials because of effective pharmacodynamic activity in a small number of healthy participants and patients with heart failure and chronic cough. Further examinations may provide therapeutic benefits for patients suffering from pain, macular edema, dermatological disorders, hydrocephalus, spinal cord injury, and cancer. There is still great interest in the development of TRPV4 antagonists. Between 2015 and 2020, 28 patent applications were received from 12 different organizations covering 8 unique chemotypes [141]. Further clinical studies are necessary to determine whether the therapeutic potential of TRPV4 antagonists can be utilized for patients.

### 2.3. Transient Receptor Potential Melastatin 8 (TRPM8)

#### 2.3.1. Brief Description of TRPM8 and Its Involvement in Pain and Headache

The TRPM8 is a nonselective cation channel with modest calcium permeability and is activated by cold temperatures (8–28 °C), membrane depolarization, menthol, and icilin [142].

**Figure 5:**
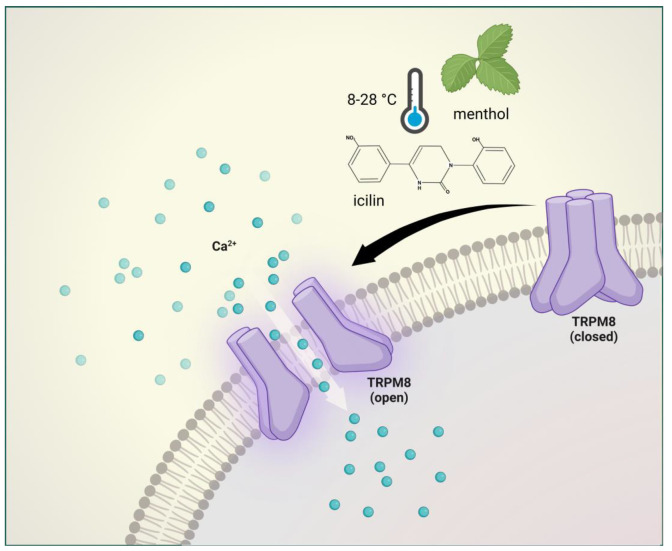
Transient receptor potential melastatin 8 receptor activation. The TRPM8 is activated by cold temperatures (8–28 °C), membrane depolarization, menthol, and icilin. TRPM8 mediates normal thermosensation and has a role in both cooling-mediated analgesia and cold hypersensitivity.

TRPM8 is expressed on C- and Aδ- sensory nerve fibers, as well as DRG and TG neurons [143]. A subset of TRPM8-positive cells may coexpress TRPV1 and/or CGRP [144,145,146]. Furthermore, it is present in hypothalamic and hindbrain nuclei responsible for autonomic thermoregulation [147]. In addition, TRPM8 is also expressed in macrophages. Activation of TRPM8 on macrophages increases the release of interleukin 10 (IL-10) and decreases the release of tumor necrosis factor (TNF), thereby causing an anti-inflammatory response [148] (Figure 2 and Figure 5).

The TRPM8 has been shown to play a major physiological role in inflammation, thermoregulation, itch, and migraine [149,150,151,152]. In addition, TRPM8 mediates normal thermosensation and has a role in both cooling-mediated analgesia and cold hypersensitivity after injury [145,153]. TRPM8 has been identified in several genome-wide association studies (GWAS) as one of the migraine susceptibility genes [154,155]. There is an association between migraine incidence and single nucleotide polymorphisms located near the TRPM8 coding region, although this seems to be the case mainly for people of Northern European ancestry [156]. It is currently unknown how these genetic variants affect the function or expression of TRPM8 and what is their role in migraine. Furthermore, about 50% of migraine patients have cold allodynia [157], which further strengthens the role of TRPM8 in the disease.

#### 2.3.2. Preclinical Data

Since topical administration of menthol has a cooling and pain-relieving effect [158], and cold weather can trigger migraines [159], the TRPM8 channel has become a target for pain and headache research.

In animal experiments, dural administration of icilin caused facial and hindpaw allodynia in rats, indicating that activation of TRPM8 caused migraine-like behaviors. These behaviors could be blocked with TRPM8 antagonist and sumatriptan, which further confirms the role of TRPM8 in the development of migraine [160].

At the same time, the application of icilin to the face can reduce the thermal pain caused by meningitis by activating TRPM8, thus able to alleviate thermal allodynia [161].

The opposite result may be explained by the fact that activation of TRPM8 causes cold pain after injury but at the same time reduces mechanical and heat pain. Therefore, the method of treating the patient can also be different based on the symptoms. TRPM8 agonists can be effective in complaints associated with mechanical hyperalgesia, while TRPM8 antagonists can be effective in cold hyperalgesia [56].

In an animal model of chronic migraine, acute mechanical hypersensitivity induced by repeated NTG administration was more persistent in females than in males, consistent with the gender dimorphism observed in migraineurs. This faster recovery in males is likely due to the testosterone-activated receptor TRPM8, which confers antinociception in a sex-dependent manner [162]. This theory is supported by the fact that testosterone administration reversed TRPM8-mediated antinociception in females. The authors believe that repeated noxious insults contribute to the persistence of the pain response in females, while males recover more quickly and are able to restore their normal sensitivity.

#### 2.3.3. Clinical Data

Several human studies suggest a role for TRPM8 in mediating pain relief [56]. Closely related to understanding the potential role of TRPM8 in promoting pain relief is one of its main agonists, menthol. Menthol is a common ingredient in topical creams that have long been used to reduce pain and provide a cooling sensation [56,163]. In a clinical trial with healthy volunteers, menthol was applied topically to the left thigh to measure skin-cooling effects using a digital infrared camera as well as self-reports on a visual analog scale. Menthol significantly decreased skin temperature compared to baseline for at least one hour [164]. In a study by Andersen et al., injection of the TRPA1 agonist cinnamic aldehyde into the forearm resulted in significant pain and neurogenic flare. However, when used together with menthol, the pain was reduced, and the mechanical pain threshold increased [165]. In a case report, after long-term administration of the chemotherapy bortezomib, neuropathic pain developed, but topical application of 0.5% menthol cream resulted in significant improvement in the response to mechanical stimuli above the threshold and reduced the degree of pain [166]. In another case, after postherpetic neuralgia, a patient suffered from severe allodynia, but the use of menthol oil at a concentration of 2 or 10% resulted in significant relief of symptoms [167].

Thus, TRPM8 may be considered a potential target for personalized medicine, and the role of menthol may be important in migraine therapy.

### 2.4. Transient Receptor Potential Ankyrin 1 (TRPA1)

#### 2.4.1. Brief Description of TRPA1 Channel and Role in Pain and Headaches

TRPA1 is a nonselective cation channel with an inward depolarizing current due to Na^+^ and Ca^2+^ ions [168]. TRPA1 channels play a role in the detection of pungent or irritating substances, such as allyl isothiocyanate (mustard oil), allicin, and diallyl disulfide (garlic) [169,170]. Moreover, gingerol (ginger), eugenol (cloves), carvacrol (oregano), and thymol (thyme) can also activate this receptor [171,172,173]. There are conflicting results that mechanical stimuli and noxious cold (<17 °C) also affect TRPA1 function [174,175]. In addition, evidence suggests that bradykinin and prostaglandins can indirectly activate TRPA1 by the activation of kinase proteins and second messengers [176,177].

**Figure 6:**
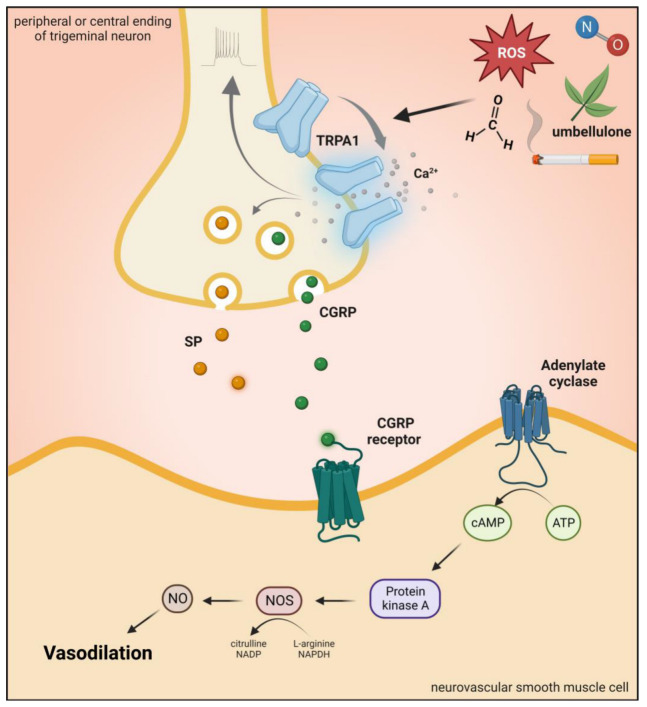
Transient receptor potential ankyrin 1 receptor activation. Activation of TRPA1 in sensory neurons induces an increase in Ca^2+^ and leads to the release of the neuropeptide CGRP, SP, and NOS-derived NO, thus mediating vasodilation. TRPA1: transient receptor potential ankyrin 1 receptor, CGRP: calcitonin gene-related peptide, SP: substance P, ROS: reactive oxygen species.

It is present in subpopulations of primary sensory neurons of the DRG, TG, and vagal ganglia (VG) [174]. TRPA1 is mainly expressed in unmyelinated C-fibers and thinly myelinated Aδ-fibers [174]. Although TRPA1 is mainly located in nociceptive neurons of the PNS, it is also found at different sites of the CNS, such as in the cortex, caudate nucleus, putamen, globus pallidus, substantia nigra, hippocampus, cerebellum, amygdala, and hypothalamus [50]. In primary sensory neurons, TRPA1 is coexpressed with SP, CGRP, and TRPV1 in primary sensory neurons, and after neuronal activation, the release of these peptides produces neurogenic inflammation and vasodilatation in the dura [169,174,178,179] (Figure 2 and Figure 6).

#### 2.4.2. Preclinical Data

Several migraine triggers can activate TRPA1, and some medications already used to treat migraine can desensitize or inhibit TRPA1 [180,181,182].

Acrolein can act on sensory nerve endings and cause neurogenic inflammation through activation of TRPA1 channels [183,184]. Several studies found that the TRPA1 response was enhanced during the acute inflammatory response provoked by Complete Freund’s adjuvant [185,186,187,188].

Glyceryl trinitrate, which can induce migraine attacks in patients, promotes NO release, which triggers pain-like responses through TRPA1 [77]. Reactive oxygen species (ROS), which are also involved in the pathomechanism of migraine and may mediate CSD, can activate TRPA1 [189]. Jiang et al. demonstrated that i.c.v. administration of anti-TRPA1 antibody can prolong the CSD latency and decrease the number of CSDs [189]. In a rodent model, TRPA1 activation leads to ROS-induced CGRP release in the TG and the dura mater [190]. It is assumed that the activation of TRPA1 in the trigeminal nerve endings in the nasal mucosa when irritating substances are inhaled causes headaches [191]. In an animal model, intranasal administration of umbellules, the compound of Umbellaria California, evokes TRPA1-mediated and CGRP-dependent neurogenic meningeal vasodilation [190,192]. Hydrogen sulfide, a gas stimulant of TRPA1, is involved in the regulation of many physiological functions and may play an important role in central nociceptive processing, causing the sensation of headache [193].

Behavioral studies suggest that TRPA1 located at the central terminal of the primary afferent ending may play a role in maintaining pain hypersensitivity and regulates transmission to glutamatergic and gamma-aminobutyric acid (GABA)-ergic interneurons [194,195]. Intraplantar injection of 4-hydroxynonenal or cinnamaldehyde (selective agonists) can activate TRPA1 and cause cutaneous neurogenic inflammation in rodents [196,197,198]. In another in vivo animal experiment, topical administration of selective TRPA1 agonists in vivo was found to induce nociceptive behavior [171]. Local injection of formalin (a TRPA1 agonist) induced acute pain and prolonged tactile allodynia at the injection site. Intrathecal administration of a low-dose TRPA1 antagonist reduced the tactile allodynia but not acute pain behavior [199]. In animal model of diabetes, topical administration of streptozotocin caused TRPA1-dependent polymodal hyperalgesia, while after systemic administration, acute sensory loss was observed [200].

However, preclinical evidence suggests that the agonism of TRPA1 may contribute to the analgesic effect induced by acetaminophen. The reactive metabolite of acetaminophen can decrease pain behavior due to the desensitization of sensory neurons containing TRPA1 [201]. This result suggests that TRPA1 agonism may represent a new approach to the development of analgesic compounds.

Several behavioral findings show that pain hypersensitivity is reduced by TRPA1 channel antagonists in various animal models. In animal models of inflammatory and neuropathic pain, the orally administered HC-030031—TRPA1 receptor antagonist—can inhibit mechanical hypersensitivity [187]. These results support that TRPA1 plays an important role in nociceptive transmission.

In addition, TRPA1 polymorphism has been linked to migraine generation [178].

#### 2.4.3. Clinical Data

There have been promising but controversial results regarding the therapeutic potential of the TRPA1 receptor in the treatment of asthma, chronic obstructive pulmonary disease, and chronic cough [202,203].

A laser Doppler imaging study demonstrates that cinnamaldehyde causes robust axon reflex flare vasodilation in humans. Moreover, it simultaneously causes a feeling of itching in some of the participants through cinnamaldehyde-induced mast cell degranulation or direct activation of itch fibers via TRPA1 [204].

Hydra Biosciences/Cubist Pharmaceuticals reported a successful Phase I trial with a TRPA1 antagonist, CB-625. However, due to its low solubility, the experiments were stopped.

Another Phase I study looked at ODM-108, a potent TRPA1 antagonist. This study was stopped due to complex pharmacokinetics. It should be noted that there were no safety concerns with this antagonist.

To date, only one TRPA1 antagonist, GRC1753689, has completed Phase II clinical trials. Good results were obtained with GRC1753689 in a Phase II trial where patients suffered from peripheral diabetic neuropathy and asthma, but it has some pharmacokinetics problems, so it cannot progress into Phase III [62].

## 3. Conclusions

**Figure 7:**
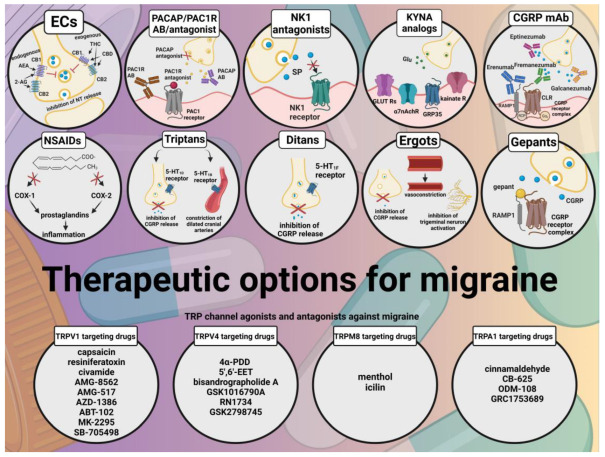
Summary of current and emerging therapeutic options for migraine. Several medications have been designed to treat migraines. Classes of medications used for acute treatment of migraine include simple analgesics such as NSAIDs; migraine-specific treatments, such as serotonin receptor agonists (triptans, ditans); and ergot derivatives. In addition to these, treatments that inhibit the release of CGRP are also receiving increasing attention: small molecule CGRP receptor antagonists and monoclonal antibodies. Future therapies include targeting additional molecules involved in the pathomechanism of migraine, such as PACAP, ECs, and NK1. Furthermore, molecules targeting the kynurenine system are also at the center of attention in migraine therapy research. As TRP channels have been repeatedly implicated in the disorder, including TRPV1, TRPV4, TRPM8, and TRPA1, modulation of these receptors may provide future therapeutic options for migraine sufferers. Red cross means inhibition.

In the last decade, studies exploring the possible mechanisms of migraine have increased exponentially. The fact that some TRP receptors are present in nociceptors and brain regions that play a role in primary headaches has provided new insights into the pathomechanism of migraine. Although drugs targeting TRP channels are not yet included in the therapy of primary headaches, preclinical studies support the role of these channels in these diseases and reinforce the importance of the further development of new and safer therapies based on the modulation of TRP channels (Figure 7). New advances in preventive therapy are essential to reduce the personal and socio-economic burden of migraine.

## Data Availability

Data sharing not applicable.

## Abbreviations

4α-PDD	4α-phorbol-12,13-didecanoate
5-HT	serotonin
BBA	bisandrographolide A
CAMKII	calmodulin-dependent kinase II
CGRP	calcitonin gene-related peptide
COPD	chronic obstructive pulmonary disease
CSD	cortical spreading depression
DRG	dorsal root ganglion
GABA	gamma-aminobutyric acid
GWAS	genome-wide association studies
IASP	International Association for the Study of Pain
IL-10	interleukin 10
KYNA	kynurenic acid
NGF	nerve-growth factor
NI	neurogenic inflammation
NMDA	N-methyl-D-aspartate receptor
NO	nitrogen oxide
NSAIDs	nonsteroidal anti-inflammatory drugs
NTG	nitroglycerin
MOH	medication overuse headache
PACAP	pituitary adenylate cyclase-activating peptide
PAG	periaqueductal gray matter
PAR2	protease-activated receptor 2
PGs	prostaglandins
PKC	protein kinases C
PKA	protein kinases A
PNS	peripheral nervous system
ROS	reactive oxygen species
RTX	resiniferatoxin
SP	substance P
TG	trigeminal ganglion
TNF	tumor necrosis factor
TRP	transient receptor potential
TRPV1	transient receptor potential vanilloid-1 receptor
TRPV4	transient receptor potential vanilloid-4 receptor
TRPM8	transient receptor potential melastatin 8
TRPA1	transient receptor potential ankyrin 1
VG	vagal ganglia
